# #Utviklingsklar: a club-based intervention to develop plans and practices for injury prevention in youth sport—acceptability, practicality, possibilities and challenges among club leaders, coaches and workshop leaders

**DOI:** 10.1136/bmjsem-2025-002766

**Published:** 2025-11-11

**Authors:** Solveig Hausken-Sutter, Christian Moen, Emilie Scholten Sjølie, Christian Thue Bjørndal, Grethe Myklebust, Merete Møller, Hege Grindem

**Affiliations:** 1 Oslo Sports Trauma Research Center, Norwegian School of Sports Sciences, Oslo, Norway; 2 Department of Sport and Social Sciences, Norwegian School of Sports Sciences, Oslo, Norway; 3 Child and Youth Sport Research Center, Norwegian School of Sports Sciences, Oslo, Norway; 4 Department of Sports Medicine, Norwegian School of Sports Sciences, Oslo, Norway; 5 Center for Muscle and Joint Health, Department of Sports Science and Clinical Biomechanics, The Faculty of Health Sciences, University of Southern Denmark, Odense, Denmark

**Keywords:** intervention, feasibility, qualitative

## Abstract

Effective youth sport injury interventions exist, but their use in applied settings is limited by low coach adherence and lack of contextual adaptation. To address this, the #Utviklingsklar intervention was developed, a club-based intervention for youth handball and football, based on interdisciplinary programme theory and collaboration with sport organisation partners and user groups (club leaders, coaches, parents, players). The intervention supports coaches and club leaders in developing team-specific plans to improve injury-preventive practices for warm-up, strength training and pain and injury management.

This qualitative feasibility study, based on observation and interview data, examines the acceptability, practicality, possibilities and challenges of #Utviklingsklar’s e-learning and workshop activities. Three workshops led by physiotherapists were observed, involving 23 participants (17 coaches, three club leaders and three workshop leaders). Subsequently, 15 participants consented to be interviewed, comprising 10 coaches, two club leaders and three workshop leaders. Data were analysed using deductive thematic analysis principles.

The e-learning was found to be user-friendly, accessible and relevant, while the workshop was valued for fostering discussions and collaboration around team-specific injury prevention plans and practices. However, some coaches sought more tailored content (ie, topics related to their own specific training sessions and players), and workshop leaders expressed a need for additional preparation and practice time to feel confident in their roles. Improved communication of #Utviklingsklar’s flexible approach, along with enhanced preparation and practice for workshop leaders in supporting coaches’ and club leaders’ development of team-specific plans and practices, is needed to enhance understanding and engagement across clubs.

**WHAT IS ALREADY KNOWN ON THIS TOPIC**

- Current injury prevention interventions have proven effective in controlled trials but face challenges in applied settings.
- Existing programmes focus solely on a biomedical perspective, often overlooking the coaches’ role, player-environment interactions and the need for contextual adaptation.

**WHAT THIS STUDY ADDS**

- This study provides insight into how a club-based intervention combining e-learning and workshops can foster collaboration and reflection among coaches and club leaders to strengthen team-specific injury prevention practices.
- Through qualitative exploration, the study captures nuanced feedback on possibilities and challenges in implementing a sport injury prevention intervention.
- The findings highlighted the need for clearer communication of the intervention’s flexible design and better training for workshop leaders to support coaches and club leaders in developing plans and practices.
- These insights directly informed refinements of #Utviklingsklar in preparation for future evaluation.

**HOW THIS STUDY MIGHT AFFECT RESEARCH, PRACTICE OR POLICY**

- This study suggests an approach for developing club-based interventions that balance consistency with flexibility, which can enhance their relevance in real-world settings.
- This study advances the field of youth sport injury prevention by addressing implementation challenges and paving the way for more adaptable, sustainable and contextually relevant interventions.

## Introduction

 Current injury prevention interventions among youth athletes have shown success in controlled trials. Examples include neuromuscular training and warm-up in young handball and football players,^1 2^ and exercise including balance, core stability, hamstring eccentrics, running/sprinting for young male football players.^3^ However, bridging the gap to applied settings remains a major challenge. Studies in Norwegian adult handball and football, as well as in Danish, Swedish and UK youth sports, reveal that only 13%–46% of coaches adhere to injury prevention interventions (eg, neuromuscular warm-ups) as intended, with reluctance to maintain use beyond the study period.^4^,^8^ Many injury prevention interventions also overlook the complex player-environment interaction, focusing primarily on biomedical elements (eg, neuromuscular training) rather than interpersonal and contextual dynamics.^9^,^11^ These findings suggest the need to move beyond one-size-fits-all approaches to injury prevention, and for interventions to be adaptable to the unique interactional player environments, cultures and youth sport demands.^12^ Such flexibility can enhance programme relevance and support long-term use. A dynamic iterative development process—grounded in interdisciplinary collaboration with sports organisations and end-users—is essential to ensure that programmes remain practical, meaningful and contextually appropriate.^13^,^15^ By integrating multiple scientific perspectives and the lived realities of youth sports, injury prevention interventions can better address the complexities of real-world application.

To address these challenges, the #Utviklingsklar project was launched in 2021, guided by the Medical Research Council’s framework for development of complex health interventions.^16^ This involved formal partnerships across research and sports organisations and a theory-based approach that combined insights from sport sociology, biomedicine and health behaviour science. This interdisciplinary foundation helped establish a programme theory for youth sport injury prevention to help explain why and how the intervention could work. Through this collaborative process of integrating disciplinary knowledge with the contextual expertise of sport organisations and user groups, the #Utviklingsklar intervention was developed as a club-based intervention to develop plans and practices for injury prevention in youth sport. The full development process and intervention is described in more detail elsewhere.^17^ Importantly, coaches were the main target of the intervention, club leaders supported the coaches’ participation and physiotherapists within an existing injury prevention network delivered the workshop in the intervention.

Due to our innovative approach, we sought to explore whether club leaders, coaches and workshop leaders would accept the topics and delivery of the #Utviklingsklar intervention.^16^ We therefore conducted a qualitative feasibility study, which enables necessary adjustment prior to full-scale evaluation.^18^ Qualitative methods are particularly suited for feasibility studies^19^ as they offer in-depth understanding of context and participant experience and can uncover barriers not captured by quantitative measures.^16^ Hence, this study aims to assess the feasibility of #Utviklingsklar intervention, with a specific focus on four key aspects: acceptability, practicality, possibilities and challenges.^18 20^

## Materials and methods

### Context

#Utviklingsklar was developed through a theory-based, interdisciplinary approach grounded in biomedicine, sports sociology and health behaviour. The intervention was co-developed with coaches, players, parents, club leaders and sports organisation partners (the Norwegian Football Association, the Norwegian Handball Federation and the Norwegian Olympic Committee and Confederation of Sports). The result is a club-based intervention where coaches develop team-specific plans to improve their practices for injury prevention, specifically warm-up and strength training as well as managing players with pain and injuries. The intervention includes three activities: (a) an e-learning course, (b) a workshop and (c) a mid-season club meeting (figure 1).

**Figure 1:**
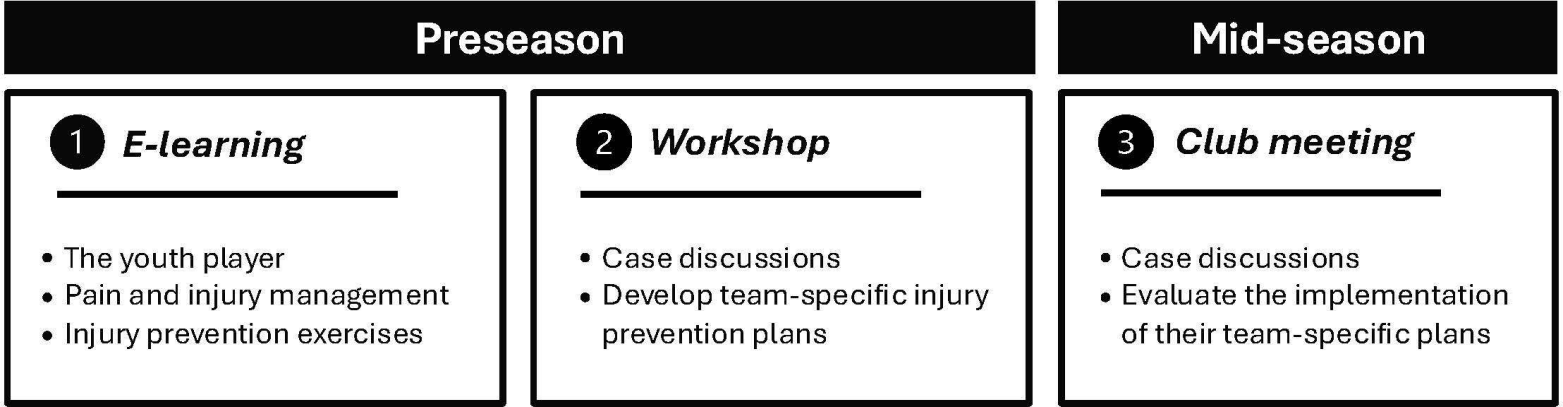
Structure and timing of the #Utviklingsklar intervention. The intervention consists of three sequential activities delivered across preseason and mid-season periods: (1) a three-part e-learning course covering youth player development, pain and injury management and injury prevention exercises; (2) a workshop focused on case-based learning and developing team-specific injury prevention plans and (3) a mid-season club meeting evaluating implementation of team-specific injury prevention plans. Each club participates with one designated leader and two to four youth team coaches.

The e-learning, designed for individual completion in about 1 hour, includes short, prerecorded video lectures ranging from approximately 0:49 to 6:50 min, interactive quizzes and module-specific questions. It also provides downloadable PDFs and web resources on managing injuries, supporting medical follow-up, reintegrating players into training, maintaining throwing loads during breaks (handball only) and applying injury prevention principles that form the basis for team-specific injury prevention plans during the workshop.

In the workshop, led by physiotherapists, coaches and club leaders work in club-based groups. First, participants discuss a case related to pain and injury management and injury prevention training. Then, using a digital form and guided discussion, coaches develop team-specific injury prevention plans outlining actions, challenges and strategies for implementation. The plans are emailed to coaches for follow-up during the season.

The mid-season meeting provides a forum for coaches and club leaders to exchange experiences with implementation. This study evaluated the feasibility of the e-learning and workshop activities.

### Study setting and participants

The study took place in Norway and included football and handball clubs with coaches who train youth players aged 13–17 years and their club leaders. A purposeful convenience sample of two football clubs and one handball club was selected based on the clubs’ location, willingness to participate and potential to offer valuable insights into youth football and handball contexts. Two clubs were characteristic of the grassroots level, while the third represented an academy club. Initial contact was made by local representatives from the project’s sport organisation partners via email or phone. On receiving confirmation from the club leader, a contact person was appointed to assist in identifying and inviting a minimum of four coaches and one club leader to form a convenience sample. This number was chosen to support the workshop format, enabling group discussions and intraclub dialogue. From football, 13 coaches and two club leaders agreed to participate and gave informed consent. In handball, four coaches and one club leader agreed to participate and gave informed consent. The coaches and club leaders completed the e-learning, attended the workshop and took part in either a group or individual interview.

A purposeful sample of three workshop leaders, who were physiotherapists, was recruited from well-established sports injury prevention networks in Norway (Skadefri and the Norwegian Sports Medicine Centre (Idrettens helsesenter)). These professionals were selected for their ability to facilitate communication and collaboration with clubs—an essential aspect of #Utviklingsklar. They also brought experience from conducting workshops for youth athletes across various sports, including football, handball and alpine skiing. The workshop leaders were approached via phone and email. All workshop leaders participated in a training day with the research group as well as completed the e-learning course, led one workshop each and participated in an individual interview following the workshops.

In total, 23 participants agreed to participate in the study: 17 coaches, three club leaders and three workshop leaders. Sixteen of the participants were men and seven were female. All coaches had basic-level or intermediate-level coach education. From the academy club, two coaches held a Union of European Football Associations (UEFA) licence, qualifying them to coach professional teams. The participants were unknown to the research team at the outset of the study.

### Procedures

The study was designed as a qualitative feasibility study of the club-based intervention #Utviklingsklar. Field observations of workshops and semi-structured individual and group interviews were conducted with coaches, club leaders and workshop leaders. Observations and interviews were conducted by the first author (SH-S), a researcher who holds a PhD in sport science and has experience in applying qualitative methodologies, including conducting observations and interviews with youth football players and their coaches. The observations and interview guides were developed based on four key aspects relevant for feasibility studies: acceptability, practicality, possibilities and challenges (see onlinesupplemental files 1  2).^18 20^ Acceptability reflects participants’ perception of the relevance, usefulness, completeness and overall experience of the intervention; practicality concerns the extent to which an intervention can be delivered when resources, time, commitment or some combinations thereof are constrained in some way; possibilities refer to factors participants felt could motivate or support them in engaging with the intervention; and challenges related to difficulties participants experienced or anticipated in completing the intervention.^18 20^

Completion of the e-learning modules was tracked in the digital learning platform, while attendance at the workshops was documented through field observation notes.

#### Observations

Participant observation with minimal interference was chosen as this method enables first-hand insights into interpersonal and contextual dynamics within a natural setting.^11 21^ Three workshops were observed, with sessions lasting between 2 hours and 2 hours and 15 min. The observations focused on how workshop leaders facilitated discussions, participant interactions and engagement with various topics. Field notes were taken on a laptop during observations to support the analysis process.^22^ These included descriptive notes (capturing what occurred, who was involved, when and where events took place and how they unfolded) as well as reflective notes (documenting emerging thoughts and ideas). Finally, the observations also served as a catalyst for the following interviews by providing contextual insights, highlighting areas for clarification and offering concrete examples that could encourage participants to reflect more deeply on their experiences.^23^

#### Interviews

Semi-structured interviews followed the observations to explore participant experiences with the e-learning and workshop, and alignment with existing practices. We opted for a semi-structured approach for its flexibility, allowing questions to be adapted based on participants’ responses, and facilitating discussion of issues arising from the observations.^24^ Individual face-to-face interviews with workshop leaders were conducted directly after the workshop session to gain in-depth perspectives on the training day, workshop and their role as facilitators.^25^ Interviews with coaches and club leaders were held 1–2 weeks later. Due to logistical issues, two football coaches participated in individual interviews instead of the planned focus groups. The interviews with the coaches and club leaders were conducted digitally (Microsoft Teams) to facilitate participation and ensure that the majority of invited participants could take part. Interviews ranged from 12 to 39 min in duration (mean=23).

### Data management and analysis

The primary data collection and management hub for this project is the University of Oslo’s services for sensitive data(TSD), compliant with the general data protection regulation (GDPR). Interviews were audio-recorded and observation logs stored electronically. Recordings were transcribed verbatim using speech-to-text software (autotekst.uio.no) and reviewed by first author (SH-S) for accuracy. Identifiable information was removed from all data to ensure confidentiality.

We analysed the interview transcript and observation notes based on the principles of thematic analysis by Braun and Clarke.^25 26^ The analytical process entailed five iterative steps, guided by the four predefined analytical aspects (acceptability, practicality, possibilities and challenges).^18 20^ A primarily semantic and deductive approach was used, which was appropriate given the study’s a priori research focus.^25^

In the first step, the first author (SH-S) familiarised herself with the data through repeated reading of interview transcripts and observation notes, accompanied by annotation and the writing of initial analytical memos. In step 2, the first author coded the material in relation to the four predefined aspects, using a semantic approach to capture explicit meanings while attending to both commonalities and subtle variations across the data.^25^

The coding process involved manual annotation of printed text and using highlighters to identify potential patterns across the data set. In step 3, the first author (SH-S) reviewed the codes and grouped them into candidate themes that reflected key patterns in participants’ experiences and practices.^26^ In step 4, these themes were refined through ongoing dialogue between the first (SH-S) and fourth author (CTB), the latter contributing methodological expertise in qualitative analysis. Finally, the entire research team engaged in collaborative discussions across iterative analytical cycles, critically reviewing and developing the thematic structure in a reflexive process to enhance depth and credibility of the findings. Through this process, the analysis was synthesised into overarching themes and subthemes, which are presented in table 1. These were then integrated with relevant data extracts to illustrate the analytic process and allow readers to assess the validity of the interpretations.^27^

**Table 1:** The overarching themes and subthemes following thematic analysis principles with supporting quotes and field notes

Overarching themes	Subthemes	Data extract from interviews and field notes to support the themes
Acceptability		
Valuing the intervention’s simplicity and knowledge	Suitable activities	*Interview*: ‘I am left with the fact that it was a useful reminder of something I may have heard before. One needs to be reminded to refresh that knowledge’. (Coach 9) *Field notes*: one coach remarked that “this [downloadable resource] is something I need to carry with me in my bag all the time!”
Questioning the topics	Irrelevant knowledge and topics	*Interview*: ‘I thought to myself, wow, they really have everything well organised. It felt, as (coach 3) also mentioned, that perhaps the material should have been pitched at a slightly higher level, since it seemed more suited for parent-coaches without established routines. The questions I received [during the workshop] were very concrete—likely not the kind of questions a parent-coach would have asked’. (Workshop leader 1)
Valuing the role as facilitators	Personally rewarding, satisfactory	*Interview*: ‘I think there were a lot of positive discussions that also made me reflect on my own role. So yes, I feel that definitely learned something from the experience’. (Workshop leader 2)
Practicality		
Practical, technical and logistical considerations	Prioritising/Finding time, log in, internet access	*Interview*: ‘Yes it [finding time for workshop] is difficult considering that we are a grassroot club and everyone works during the day, and then we have different training times in the evenings. It really comes down to prioritising’. (Coach 8)
Preparation and time constraint	Delivery methods	*Interview*: ‘After the training day, I was left with several questions, partly because I didn’t know what to expect. I felt I had very little time to process the material and prepare myself for today [the workshop]’. (Workshop leader 2) *Field notes*: the workshop leader provided limited time for discussions and appeared to guide participant’s responses too strongly.
Possibilities		
Interactive learning	Video and immediate feedback from facilitator	*Interview*: ‘It was good [the interactive elements in the e-learning]. Pedagogically well done. It made it more varied’. (Coach 8)
Collaboration and networking	A meeting space for dialogue	*Interview*: ‘I found it very valuable that the club leader participated, bringing forward concrete measures they wanted to address. It was also positive that they [coaches and club leader] were present together, as this allowed the dialogue to begin already at this stage’. (Workshop leader 2) *Field notes*: the club leader contributed with several suggestions that were discussed with the coaches. The cross-dialogue was constructive, and it was evident that the topic of warm-up requires further discussions within the club.
Personal developments, pedagogical practice	Insight into coaching practice and being outside of the clinic	*Interview*: ‘Yes absolutely. [As a physiotherapist] you see the challenges the clubs face because you are usually on the receiving end of those who are injured. So [in the workshop] you get to see it from the other side—how the clubs struggle with putting systems and routines in place. In that sense, it is very useful’. (Workshop leader 2)
Challenges		
Technical issues	Log in, inadequate internet connection	*Field notes*: one coach tells me that he/she was unable to log in to the e-learning platform due to not having BankID (Norway’s national electronic identification system used for secure online authentication and signing).
Group dynamics and participant engagement	Engagement and motivation	*Field notes*: the coaches discuss two and two, great discussions. One coach questioned why more coaches were not included in the study, noting that others had expressed interest.
Breach of expectation, uncertainty of roles	Information about the study	*Interview*: ‘…and that’s about being more prepared, or at least aware of what you are actually entering into. Clarifying expectations—that’s the word we’re looking for. That we and [project team member] had been a bit clearer about what we were actually supposed to do’. (Coach 8)
Sustainability and follow-up	Clarification of roles	*Interview*: ‘It [the workshop] was useful, but who was actually speaking to us? As far as I understood, it wasn’t a physical trainer or a football physio with extensive expertise in injury-preventive training’. (Coach 8) *Field notes*: the participants did not really reach a clear understanding of how to carry the material forward, and the rules (that they made) did not seem to be fully grasped. A more explicit conclusion or closure seems necessary.

## Results

Fourteen coaches and two club leaders completed the e-learning while all coaches and club leaders attended the workshop (17 coaches and three club leaders). All three workshop leaders completed the e-learning and facilitated one workshop each. Moreover, 15 participants provided interview data: 10 coaches, two club leaders and three workshop leaders. Participants who did not complete the e-learning reported that they had forgotten to do so, while those who did not take part in the interviews cited scheduling conflicts or technical difficulties (eg, poor internet connection).

### Acceptability, practicality, possibilities and challenges

Table 1 provides an overview of the four general aspects, overarching themes and subthemes while extracts from field notes and quotes from the interviews are brought forward in the following paragraphs to support table 1.

#### Acceptability

*E-learning:* the e-learning module was well-received by most coaches and club leaders, as one handball coach remarked: “I think that the e-learning course should be mandatory for all coaches in our club” (coach 14). Many appreciated its brevity, ease of navigation and clear presentation. While some coaches felt that the topics did not offer entirely new information, they valued its comprehensive approach and saw it as a helpful quality assurance tool for their clubs. However, a club leader expressed a desire for more specific and detailed knowledge and exercises tailored to their club’s needs, as exemplified by this excerpt:

I think it works well for the planned target group. [For us] it could have been even more specific in exercises, a more narrow and detailed focus… Maybe I would have liked to focus more specifically on individual muscle groups and delve into injury prevention at a more detailed level. (Club leader 1)

This need for specificity and tailored topics was also evident during the workshops, where some of the coaches raised several club-specific questions and concerns, particularly regarding specific injured players or those experiencing pain. Additionally, some coaches expressed uncertainty about the ‘correct’ approach and knowledge regarding injury prevention, noting differing opinions within academy clubs. These conflicting perspectives on how to handle injuries and pain led some coaches to question the topics of the e-learning and workshop, as their club had provided specific guidelines that were not always aligned with the intervention’s recommendations. For example, one coach emphasised in the interview that “You can agree or disagree with the topics presented in both the workshop and the e-learning… It is crucial to ensure that the information does not get outdated” (coach 9). This coach was hesitant to fully adopt all aspects of the intervention, while also feeling uncertain about the ‘best’ approach for injury prevention.

*Workshop:* based on the observations from the workshops, most of the coaches and club leaders were engaged and contributed with numerous ideas and suggestions for solutions and future actions. Based on the interviews, workshop leaders were pleasantly surprised by the coaches’ level of knowledge and engagement, noting, “We encountered highly reflective coaches who demonstrated considerable knowledge and familiarity with the issue. They already proposed very effective solutions” (workshop leader 1). Despite the coaches’ existing knowledge, most coaches expressed positivity and found the workshop valuable:

The workshop was excellent! It was well-organised and systematic. I particularly enjoyed the last part where we had to fill in [plans] ourselves—it made everything more concrete. Plus, the group was eager and engaged, which made it even more enjoyable. (Coach 14)

Furthermore, the coaches appreciated the different tasks they performed during the workshop, such as case work, developing team-specific injury prevention plans and applying several of the printed guidelines from the e-learning. As one coach expressed during the interview: “I really enjoyed the case assignment. Applying materials [from the e-learning] that we received to realistic situations was very useful for learning” (coach 17).

Although most coaches enjoyed the various tasks, some found the final part of the workshop—where they were asked to develop team-specific injury preventive plans—frustrating. One coach remarked, “Those implementation questions were very unclear. I didn’t know how to answer them. For example, when asked ‘how to incorporate it [the plan] into the club?’, I just wrote ‘make it into a rule’ in every column” (coach 13). This sentiment was shared by a few others, and even the workshop leaders noticed that some of the participants struggled with this part and needed more time than initially planned.

The workshop leaders were identified as crucial in conducting the workshop, responsible for guiding discussions and bridging the gap between e-learning topics and practical workshop activities. Overall, all three workshop leaders expressed appreciation for their role, describing it as valuable, enjoyable and educational. As one workshop leader stated during the interview:

A fun, exciting way to work. Very interesting to be able to convey knowledge while the participants are so involved in it themselves. And a fantastic way to underpin and support the knowledge the coaches and the club already have. Because they are the ones who know their own club best, their own conditions. (Workshop leader 3)

#### Practicality

*E-learning:* based on interview data, all coaches and club leaders found the e-learning easy to integrate into their daily routines, whether they were full-time coaches or had other full-time jobs. They thought it was quick to complete, fitted well into their schedules and required minimal resources: “The advantage is that you can do it whenever you have a free hour, in a busy everyday schedule” (coach 9). However, one coach encountered technical issues with logging in and was unable to complete the e-learning before the workshop.

*Workshop:* coaches highlighted in the interviews logistical difficulties of finding a suitable time for everyone to attend the workshop, but they emphasised that prioritising this should be a fundamental part of the club’s organising and something that the coaches ‘just do’. It was particularly challenging to find time for those who were not full-time coaches, but as one coach remarked, “It’s all about prioritising. If the leaders emphasise that this is important for us as a club and for our players, then it can and should be prioritised over a training session” (coach 5). None of the coaches or club leaders mentioned any issue related to resources (ie, providing a venue and technical equipment).

The workshop leaders faced challenges in managing time and guiding group discussions as planned. One workshop leader expressed during the interview that these issues tend to resolve with practice and acknowledged that the first time conducting such a workshop is always a bit challenging in terms of timing: “It gets a bit unfocused, so you become unsure of how much to correct. Yes, with the discussions—where should you set the boundaries? But I do find it somewhat rewarding to learn from that part” (workshop leader 1). One workshop leader noted that the feasibility study had fewer participants and smaller groups compared with what is planned for the larger study and that facilitating larger groups might be more demanding for the workshop leader, especially considering time management and organisation. In the small group setting of the feasibility study, the workshop leader could sit next to the coaches and engage directly in the discussion, but in a larger group, this would be less feasible, requiring a different approach. As the workshop leader explained: “Yes, maybe it’s the number of participants, or it might not be more demanding, but you just have to manage it differently [as a workshop leader]. That you can’t really have joint discussions but instead may need to sit in smaller groups and discuss among yourselves” (workshop leader 2).

#### Possibilities and challenges

*E-learning:* the e-learning was found to be highly accessible, with several coaches and club leaders highlighting during the interviews the convenience of completing it anytime and almost anywhere. It was also found to be flexible, allowing for self-paced learning where participants could progress through the material at their own speed. One coach noted, “There was no problem with that [completing the e-learning], and it was helpful for others to take it in smaller, divided segments” (coach 6). The e-learning was found to be interactive and engaging, featuring videos, quizzes and interactive elements designed to enhance engagement and retention. A handball coach remarked, “You clicked through and answered questions, and I received a score that caught my attention. I realised I needed to read more thoroughly to answer correctly, which made me more engaged” (coach 7). A football coach added, “That was nice [the interactive elements]. It was educationally beneficial and added variety” (coach 8).

Although the e-learning received mostly positive feedback, some participants encountered challenges such as login problems and inadequate internet connections. Another challenge was topic delivery; ensuring the material is engaging and understandable can be difficult. One coach mentioned during the interview that each e-learning module left her with more questions than answers, as she found the topics too general. Nevertheless, the e-learning was widely appreciated for its flexibility and accessibility, which allowed the participants to engage with the material at their own pace.

*Workshop:* one of the key possibilities of the workshop noted during observations and highlighted in some interviews was its ability to facilitate collaboration and networking within the club. For example, most coaches appreciated having the workshop alongside the club leaders, as one coach remarked:

In the future, when it comes to matters ‘on the floor’, it is important to involve both coaches and club leaders to understand their daily lives. This way, we can support each other and gain insights from both perspectives. So, instead of separate workshops for coaches and club leaders, having them together, as we did, is much more effective. (Coach 5)

One of the club leaders held a dual role as both coach and club leader, a common situation in many clubs. During the group interview, this individual remarked about the workshop:

I was not prepared to take on the role of the club leader, but it turned out to be very rewarding. It provided an overarching perspective on how we, as a club, can establish guidelines related to that work. It was great that this aspect was included as well, it allowed us to elevate the discussion to a club-wide level. That was very beneficial. (Club leader 3)

For workshop leaders, facilitating the session offered opportunities for personal development and pedagogical practice. In interviews, all workshop leaders emphasised the valuable insights gained through interaction with clubs, particularly in injury prevention practices. As clinicians—and in some cases, parents of young players—they found it enlightening to understand club and coach perspectives.

Logistics posed a major challenge, requiring coaches to coordinate venues and schedules around busy training calendars. Despite this, most participants remained solution oriented.

The presence of a club leader was generally seen as positive for collaboration; however, one instance revealed the opposite. In this case, the club leader expressed strong opinions about club procedures and seemed to dominate the conversation. The workshop leader’s attempts to facilitate discussion between coaches and club leaders appeared to yield only limited collaborative outcomes. Reflecting on this, the workshop leader remarked, “It is possible that there are things the coaches want to say but refrain from doing so because the club leader is present” (workshop leader 3).

Other challenges included breaches of expectations and uncertainty of roles. For example, one coach asked: “What are we participating in exactly, who is talking to us, and how can we move forward from here?” (Coach 4). Similarly, a workshop leader felt unprepared for her role, noting that she felt ‘thrown into it’. She wished for more training, especially since her role differed from her previous experiences as a workshop leader where she ‘simply read from a manuscript’. All workshop leaders also expressed uncertainty about their role as professional advisors, particularly when faced with specific and detailed questions from coaches regarding injured players and medical issues. Those who felt more prepared were better able to facilitate discussions and connect with participants.

Finally, concerns about sustainability and follow-up emerged. One coach remarked,

It is wonderful to feel wise and aware during the workshop, but we need to extend that knowledge to the club. It’s about moving forward. What I missed was a more concrete plan for how to proceed after the workshop. It’s a shame to have such a productive workshop without following through (coach 14).

## Discussion

The main finding of this study was that the topics and delivery of a club-based intervention like #Utviklingsklar was found to be acceptable and practical among a sample of club leaders, coaches and workshop leaders.

Of 17 coaches and three club leaders, 14 coaches and two club leaders completed the e-learning and all attended the workshops, which demonstrates their willingness to engage with the intervention. Most participants found the e-learning and workshop acceptable, and they were in general satisfied with the topics, format and execution. Many coaches and club leaders were actively engaged in workshop discussions and stated that they enjoyed the experience. They also highlighted the benefits of working together within the club during the workshop, as this could strengthen their relationship, which again could benefit the club’s plans and practices for injury prevention. Workshop leaders also reacted positively towards the workshop and demonstrated a willingness to engage and satisfaction with their role as facilitators. However, their challenges with managing time and facilitating group dialogue, especially as #Utviklingsklar will involve larger groups and more participants in the upcoming evaluation study, point to a key implication for future implementation: pedagogical competence develops through experience, reflection and repeated practice—elements that are well-established as essential to professional growth in educational contexts.^28^

Preseason workshops like those in #Utviklingsklar, which combine both theoretical and practical activities, have shown several positive outcomes such as boosting youth soccer coaches’ confidence in using injury prevention programmes.^29^ For example, in the study by Owoeye *et al*,^30^ coaches reported an improved confidence in understanding and implementing the programme after participating in a workshop, even when they faced challenges like player disinterest. However, research is limited on whether this increased confidence translates into long-term adoption, adherence and sustained use of the programme. Møller *et al*^31^ found that a combined online and onsite implementation strategy, which featured coach workshops and support from health professionals, did not outperform an online-only approach in improving adherence to an injury prevention exercise programme. This underscores the need for further research on implementation strategies and their effectiveness in promoting sustained programme use over time.

Although most coaches and club leaders who participated in the feasibility study enjoyed and valued the easiness of the e-learning and workshop, some coaches expressed reluctance towards the topics and delivery methods. Interviews and observations suggested that this reluctance stemmed from perceptions that the material was too general, alongside a preference for more specific exercises and a greater need for club-tailored topics. The reluctance was particularly evident during the final part of the workshop, where coaches were asked to develop injury prevention plans tailored to their own teams. Coaches who found the topics too general seemed to struggle the most with this task, possibly due to a perceived disconnect between the broad topics presented earlier and the sudden requirement for specificity (ie, make concrete and team-specific plans). The shift from general knowledge to specific tasks may have felt unfamiliar and difficult for them. Considering that this final part of the workshop is a critical component of #Utviklingsklar—where the goal is to develop team-specific plans to be implemented throughout the season—improvements are needed. For instance, better communication about the intervention’s structure and how it helps clubs enhance injury-prevention coaching practices through the development and implementation of specific plans for injury-preventive warm-up, strength training and managing players experiencing pain or injury is essential. Additionally, coaches should have a clear understanding of what is expected of them before, during and after the workshop. Finally, it is essential for #Utviklingsklar that workshop leaders are fully equipped to guide coaches and club leaders through the workshop and actively support them in developing effective, team-specific plans and practices.

The reluctance towards the topics may also stem from a mismatch in expectations, as many coaches seemed to anticipate a more ‘traditional’ injury prevention intervention, featuring the prescription of specific exercises like those found in popular programmes such as Knee Control,^32^ The 11+^2^ or Nordic hamstring exercises.^33^ These expectations reflect an underlying assumption that injuries can be prevented, at least in part, by strengthening specific muscle groups—a dominant approach within current sports injury prevention work.^11 34^ Although traditional injury prevention programmes have demonstrated effectiveness, they are developed from a strictly biomedical perspective, neglecting the significant impact of interpersonal and contextual dynamics on injury risk.^11^ To address this gap, #Utviklingsklar was co-developed with sport organisation partners, coaches and players to offer greater flexibility, contextual adaptation and autonomy for coaches—key factors that coaches and players consider as supportive for successful integration of programmes into regular practice.^35^ However, even if coaches imply that they desire more flexibility and autonomy, they may still seek clearer advice on how to implement it.

Finally, a key challenge in designing educational interventions like #Utviklingsklar lies in addressing the heterogeneity of the youth coaching population in Norway—ranging from formally educated and certified professionals to voluntary, non-certified parent-coaches—which complicates efforts to ensure topic relevance, appropriate depth and pedagogical alignment across such a diverse user group.

### Implications

Due to #Utviklingsklar’s flexibility, which allows coaches to develop plans to their own context, the intervention has the potential to be less of an ‘add on’ compared with other programmes, which can enable the integration into regular practice.^8 35^ Nevertheless, as O’Brian *et al*^12^ emphasises, it is important to strike a balance between maintaining essential and consistent elements and allowing modifications to enhance contextual fit. For the refinement of #Utviklingsklar, it is important to clearly communicate its flexible approach from the outset and strengthen workshop leader training to better support coaches in developing team-specific injury prevention plans and fostering collaboration within clubs. Flexibility, contextual adaptation and ongoing support have been shown to influence coaches’ perceived value and sustained use of injury prevention practices.^36^ However, evidence on long-term outcomes, such as reduced injury incidence or improved performance over multiple seasons in real-world settings, remains limited, which highlights the need for continued evaluation. Future research should explore how coaches integrate and maintain the plans into their teams’ regular practice, as well as how players experience #Utviklingsklar. Since traditional programmes are often criticised for being boring and misaligned with players’ primary motivations for participating in sports, such as fun and enjoyment,^8 37^ #Utviklingsklar may offer a more engaging alternative by emphasising the coach’s and club’s role in creating a positive motivational climate.^38^

### Methodological considerations

The use of qualitative methods, particularly a combination of interviews and observations in a feasibility study, is a strength as it offers valuable insights into the implementation experiences of club leaders, coaches and workshop leaders. Understanding the perspectives of all these groups is essential, as they hold key roles in the #Utviklingsklar intervention. Furthermore, conducting the observations before the interviews provided us with contextual insights, which helped to frame more relevant and informed interview questions.

There are some limitations to this study that should be noted. First, the sample lacked diversity, as it included only one handball club and two football clubs from the region of Oslo, which may not be representative of all Norwegian football and handball clubs. However, this study aimed to provide detailed, context-specific insight into intervention topics and delivery of #Utviklingsklar, and not broad generalisability of findings. Second, the initial thematic analysis was conducted by a single researcher, which may have introduced individual bias in the interpretation of the data. To enhance the credibility of the findings, reflexive practices were employed, including critical reflections with the fourth author and ongoing discussions with the research team to review and refine interpretations. Third, we did not examine what happens after the workshop, such as whether coaches attended the planned mid-season meeting or implemented their injury prevention plans. Finally, the workshop format differed from what is planned for the larger study, and as noted by workshop leaders, managing time and facilitating larger groups may present greater challenges. These aspects will be explored further in the comprehensive evaluation study.

## Conclusion

The findings from the feasibility study highlight both the strengths and areas for improvement in the #Utviklingsklar intervention. While most participants appreciated the simplicity and flexibility of the e-learning and workshop activities, some coaches expressed reluctance towards the intervention’s topics and delivery methods, likely due to expectations of a more traditional exercise-focused programme. Clearer communication of #Utviklingsklar’s flexible, context-driven approach and clarification of expectation is necessary. Additionally, improving workshop leader training is important so they can better support coaches and club leaders in developing team-specific injury prevention plans, which can enhance understanding, engagement and practical application.

## Supplementary material

10.1136/bmjsem-2025-002766 online supplemental file 1

10.1136/bmjsem-2025-002766 online supplemental file 2

## Data Availability

Data are available on reasonable request. No data are available.
