# Supplementary material for: #Utviklingsklar: a club-based intervention to develop plans and practices for injury prevention in youth sport—acceptability, practicality, possibilities and challenges among club leaders, coaches and workshop leaders
Source: BMJ Open Sport Exerc Med. 2025 Nov 11;11(4):e002766. doi: 10.1136/bmjsem-2025-002766 (PMC12606477; doi:10.1136/bmjsem-2025-002766)
Supplement: online supplemental file 1 [file bmjsem-11-4-s001.pdf]

Interview guide – coaches and club leaders

Part 1 – Introduction

- Welcome
    - *Purpose of the interview*
- Anonymity
    - *Clarify permission to record the interview*
- Introduction of participants
    - *Can you tell me a little about your background and what you do today?*

Part 2 – Acceptability

E-learning

- What are your main takeaways after completing the e-learning?
- Was there anything in the e-learning that was relevant to your practice?
- Was there anything in the e-learning that was new?
- Was there anything missing from the e-learning?

Workshop

- What are your main takeaways after completing the workshop?
- Was there anything in the workshop that was related to your practice?
- Was there anything in the workshop that was new?
- Was there anything missing from the workshop?
- Hvordan opplevde dere kombinasjonen e-læring etterfulgt av workshop?

Part 3 – Practical considerations

- Do you see any practical challenges with the e-learning?
- Do you see any practical challenges with the workshop?
- How can we make it easier for you to complete the e-learning and workshop in this project and in the future?

Part 4 – Possibilities and challenges

- What did you find challenging about completing the program?
- What could motivate you to complete the program?

Part 5 – Conclusion

- Have you started to put into practice any of what you went through in the program since last time?
- Is there anything else you would like to add before we finish?
