# Supplementary material for: #Utviklingsklar: a club-based intervention to develop plans and practices for injury prevention in youth sport—acceptability, practicality, possibilities and challenges among club leaders, coaches and workshop leaders
Source: BMJ Open Sport Exerc Med. 2025 Nov 11;11(4):e002766. doi: 10.1136/bmjsem-2025-002766 (PMC12606477; doi:10.1136/bmjsem-2025-002766)
Supplement: online supplemental file 2 [file bmjsem-11-4-s002.pdf]

Interview guide – workshop leaders

Part 1 – Introduktion

- Welcome
  - *Purpose of the interview*
- Anonymity
  - *Clarify permission to record the interview*
- Introduction of participant
  - *Can you tell me a little about your background and what you do today?*

Part 2 – Training day

First, we will talk about the training you received at NIH before the workshop.

Acceptability - training

- What are your takeaways after completing the training?
- Was anything in the training relevant to what you have done previously as an ambassador/workshop leader?
- Was there anything new in the training?

Practicality - training

- Do you see any practical challenges with the training?
- How can we make it easier for ambassadors like you to complete the training in the future?

Time use
Amount of preparation/time required

Possibilities and challenges – training

- What kind of possibilities do you see personally in taking part in the training and workshop?
- What challenges did you experience in connection with the training?

Part 3 – Workshop

Now we will talk about the workshop you carried out with the club

Acceptability - workshop

- What are your takeaways after completing the workshop?
- Were any of the topics in the workshop relevant to what you have done previously?
- Were there any new topics in the workshop?
- How was it to work with the case tasks in the workshop?
- How was it to work with the development of team-specific injury prevention plans?
- How was it to take on the role of facilitator?

Practicality - workshop

- Do you see any practical challenges with conducting the workshop?
- How can we make it easier for ambassadors like you to run the workshop in this project and in the future?

Possibilities and challenges - workshop

- What possibilities do you see personally in taking part in the training and workshop?
- What challenges did you experience regarding the training and workshop?

Part 4 – Conclusion

- Is there anything else you would like to add before we finish?
